# Supplementary material for: Characterising the Gene Expression, Enzymatic Activity and Subcellular Localisation of Arabidopsis thaliana Metacaspase 5 (AtMCA-IIb)
Source: Biology (Basel). 2023 Aug 22;12(9):1155. doi: 10.3390/biology12091155 (PMC10525968; doi:10.3390/biology12091155)
Supplement: Supplementary file 1 [file biology-12-01155-s001.zip › biology-2533452-supplementary.pdf]

Supplementary Materials-

Original gels and blots for Figure 6 (A, B, D)

Figure 6 A

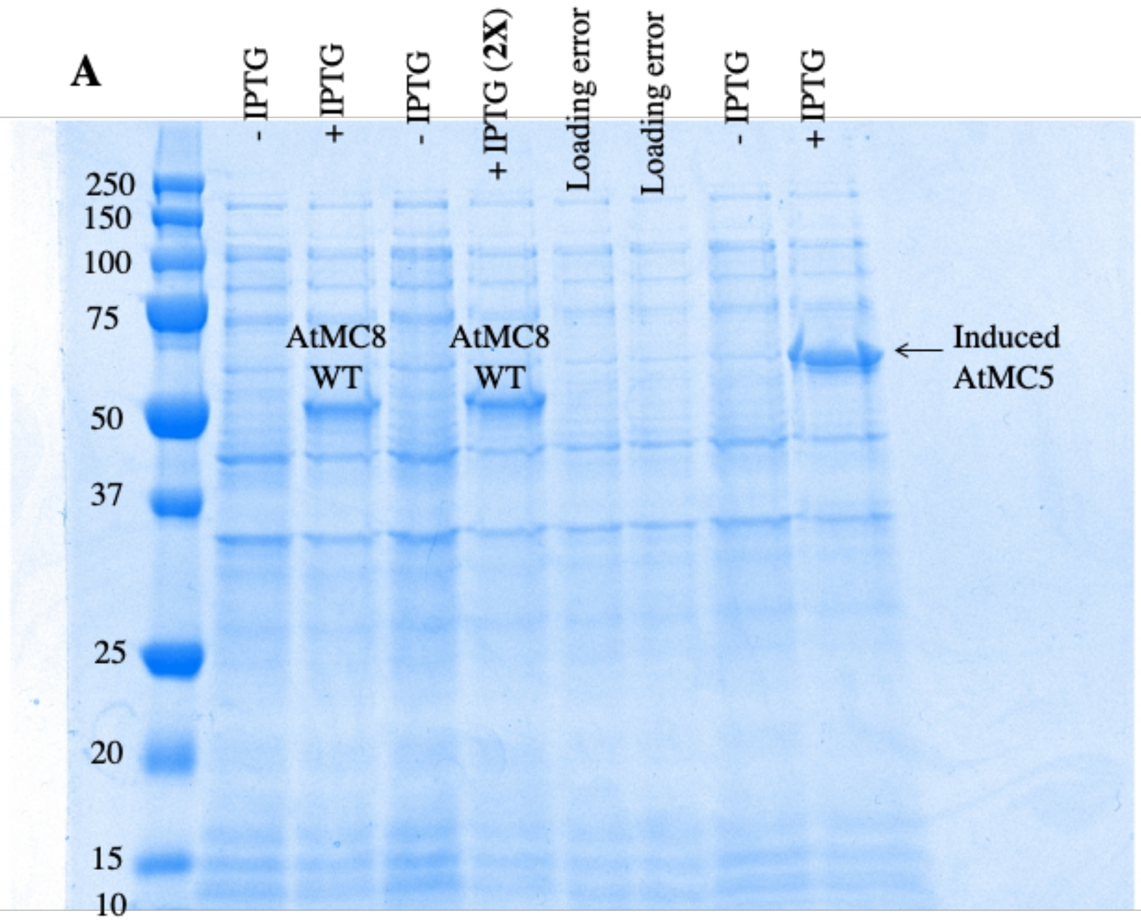

Figure S1: 6A **Expression of recombinant AtMC5 wild type in *E. coli*.** Crude extract from bacterial cultures containing IPTG (+IPTG) and no IPTG (-IPTG) was separated by SDS-PAGE and stained with Instant blue. Recombinant AtMC8 was run as a control for expression.

Figure 6 B

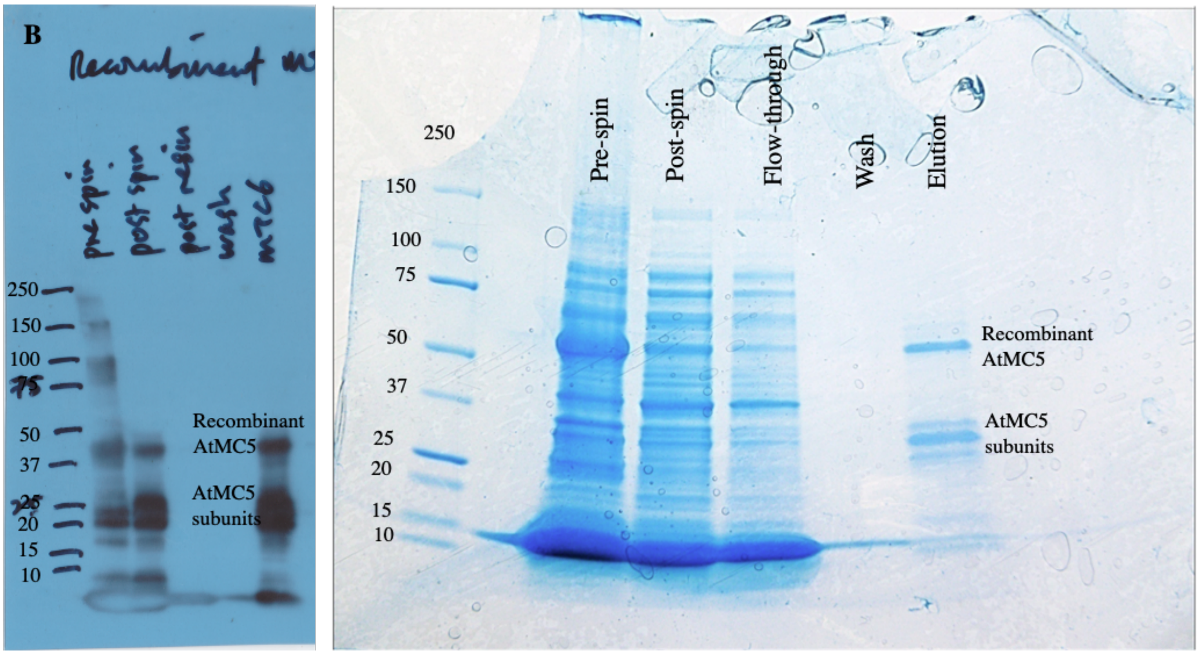

Figure S2: 6B **Expression of recombinant AtMC5 wild type in *E. coli*.** Recombinant AtMC5 wild type was purified using a His-tag affinity resin. Left panel: A western blot was analysed using anti-his (1:3000) as primary antibody and anti-mouse (1:3000) as secondary antibody. Right panel: The purified fraction was also separated through SDS-PAGE and stained with Instant Blue.

Figure 6 D

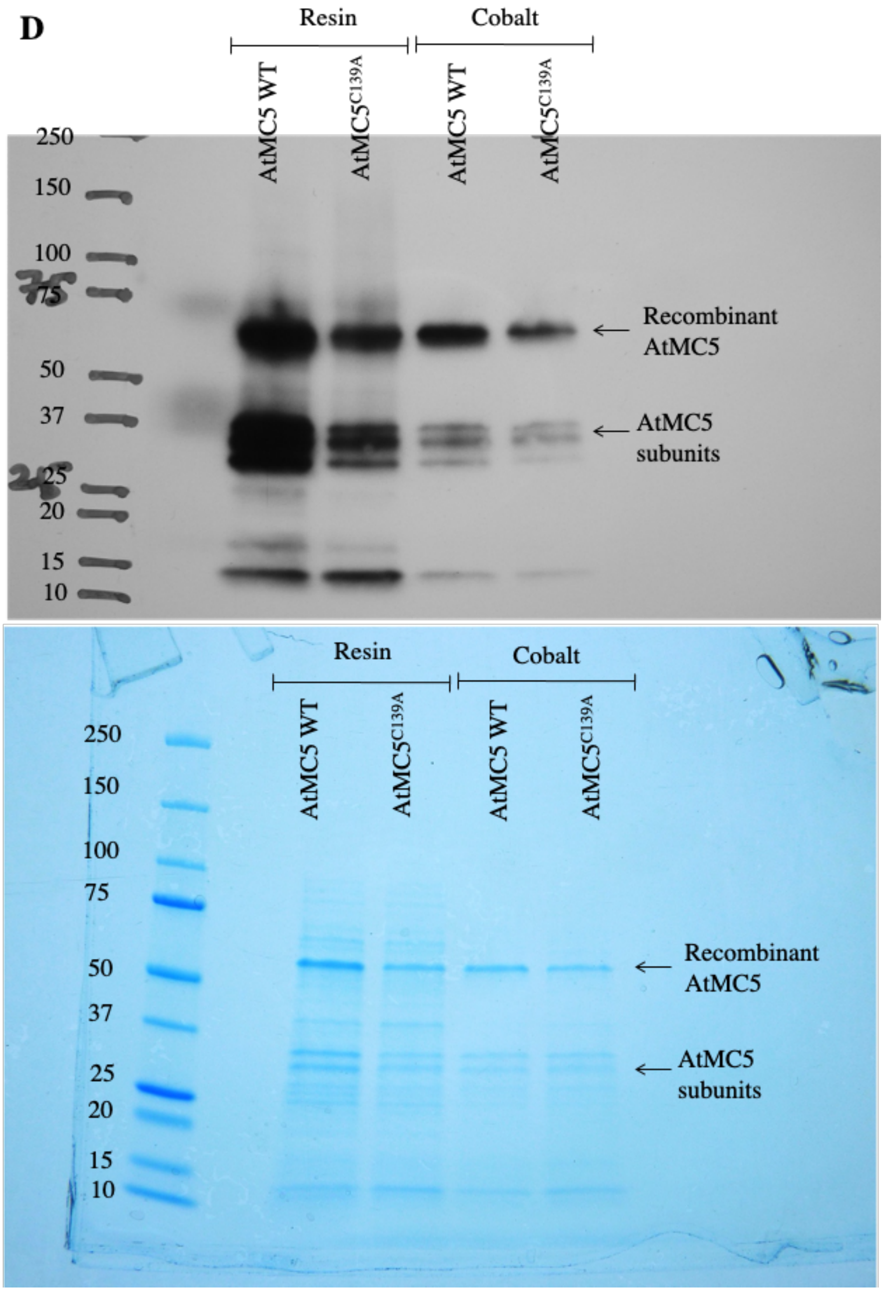

Figure S3 : 6D-**Expression of recombinant AtMC5 wild type and C^{139A} in *E. coli*.** Mutation in the catalytic site of AtMC5 was introduced at position C139A. Recombinant AtMC5 wild type and mutant C139A was purified using a His-tag affinity resin and cobalt. A western blot was run using anti-his (1:3000) as primary antibody and anti-mouse (1:3000) as secondary antibody (higher panel). The purified fraction was also separated using SDS-PAGE and stained with Instant Blue (lower panel).
